# Reconstruction of large segmental bone defects in rabbit using the Masquelet technique with α-calcium sulfate hemihydrate

**DOI:** 10.1186/s13018-019-1235-5

**Published:** 2019-06-26

**Authors:** Zhu Long Meng, Zi Quan Wu, Bi Xin Shen, Hong Bo Li, Yang Yang Bian, De Lu Zeng, Jian Fu, Lei Peng

**Affiliations:** 1 Municipal Hospital Affiliated to Medical School of Taizhou University, Taizhou, China; 2 Department of Trauma Center, The First Affiliated Hospital of Hainan Medical University, Haikou, China; 3 School of Pharmaceutical Sciences, Wenzhou Medical University, Wenzhou, China

**Keywords:** Bone defect, The Masquelet technique, α-calcium sulfate hemihydrate

## Abstract

**Background:**

Large segmental bone defects can be repaired using the Masquelet technique in conjunction with autologous cancellous bone (ACB). However, ACB harvesting is severely restricted. α-calcium sulfate hemihydrate (α-CSH) is an outstanding bone substitute due to its easy availability, excellent biocompatibility, biodegradability, and osteoconductivity. However, the resorption rate of α-CSH is too fast to match the rate of new bone formation. The objective of this study was to investigate the bone repair capacity of the Masquelet technique in conjunction with isolated α-CSH or an α-CSH/ACB mix in a rabbit critical-sized defect model.

**Methods:**

The rabbits (*n* = 28) were randomized into four groups: sham, isolated α-CSH, α-CSH/ACB mix, and isolated ACB group. A 15-mm critical-sized defect was established in the left radius, followed by filling with polymethyl methacrylate spacer. Six weeks after the first operation, the spacers were removed and the membranous tubes were grafted with isolated α-CSH, isolated ACB, α-CSH/ACB mix, or none. Twelve weeks later, the outcomes were evaluated by manual assessment, radiography, and spiral-CT. The histopathological and morphological changes were examined by H&E staining. The levels of alkaline phosphatase and osteocalcin were analyzed by immunohistochemistry and immunofluorescence staining.

**Results:**

Our results suggest that the bone repair capacity of the α-CSH/ACB mix group was similar to the isolated ACB group, while the isolated α-CSH group was significantly decreased compared to the isolated ACB group.

**Conclusion:**

These results highlighted a promising strategy in the healing of large segmental bone defect with the Masquelet technique in conjunction with an α-CSH/ACB mix (1:1, *w*/*w*) as they possessed the combined effects of sufficient supply and low resorption.

## Background

The treatment of large segmental bone defects caused by acute high-energy trauma, tumor destruction or resection after infection remains challenging for most orthopedic and trauma surgeons [1]. The approaches currently used to treat large segmental bone defects including autologous bone graft, vascularized free bone transfer, and the Ilizarov intercalary bone method [2]. Autologous bone graft remains the gold standard treatment for bone defects less than 5 cm because of their osteogenicity, osteoinductivity, and osteoconductivity [3–5]. Vascularized free bone transfer [6–8] and the Ilizarov intercalary bone method [9, 10] are used most commonly to treat large segmental bone defects more than 5 cm. However, vascularized free bone transfer is microsurgically demanding. In addition, there are associated with donor site morbidity and a high failure rate [11]. The Ilizarov intercalary bone method is technically demanding, time-consuming, and associates with a high complication rate [12].

The Masquelet technique, an emerging alternative approach first described by Alain Masquelet [13, 14], consists of a two-stage procedure that allows reconstruction of large segmental bone defects of up to 25 cm [15, 16]. In the first stage, the defect is temporarily filled with a polymethyl methacrylate (PMMA) spacer. The spacer serves both a mechanical and a biological role. It gives structural support, hinders fibrous invasion, and importantly, induces a vascularized membrane [17]. The induced membrane has been shown to produce growth factors and osteogenic factors, supporting the differentiation and proliferation of bone mesenchymal stem cells (BMSCs) [18, 19]. Moreover, the induced membrane inhibits soft tissue invasion of the defect, thereby potentially preventing autologous cancellous bone (ACB) resorption [4]. In the second stage, the foreign-body PMMA spacer is removed after 6–8 weeks and the membranous tube is filled with ACB [16, 20]. However, ACB is severely restricted by the scarce supply of donors. In addition, regardless of technique, ACB harvesting can also be associated with complications such as bleeding, hematoma, and infection [21–23]. For these disadvantageous reasons, the use of bone substitute would be of great interest, such as α-calcium sulfate hemihydrate.

In recent years, α-calcium sulfate hemihydrate (α-CSH), an important class of highly cementitious bone substitute, has been clinically used to treat large bone defect. Excellent biocompatibility, biodegradability, and osteoconductivity of α-CSH biomaterial make it an outstanding bone substitute with properties that are more similar to the autologous bone [24]. Interestingly, it has been found to be potentially osteoinductive like differentiation of BMSCs into osteoblasts [25]. Furthermore, the pH environment and the ion release behavior of α-CSH provide an appropriate microenvironment for bone regeneration [26, 27]. However, the resorption rate of α-CSH is too fast to match the rate of new bone formation.

Based on these studies, we found that the Masquelet technique can produce growth factors and osteogenic factors and protect ACB against degradation. On the other hand, α-CSH is a sufficient bone substitute and has excellent biocompatibility, biodegradability, osteoconductivity, and osteoinductivity. In this study, we hypothesized that by replacing or reducing ACB demanding, the combination of the Masquelet technique and isolated α-CSH or an α-CSH/ACB mix can heal large segmental bone defect, which may represent a promising bone substitute to reduce ACB demands to treat large segmental bone defect.

## Materials and methods

### Animals

All animal experiments were performed according to protocols approved by the Animal Care and Use Committee of Hainan Medical College and the Use of Laboratory Animals Guideline of the National Institutes of Health (registration number SYXK 2014-0006). Twenty-eight male New Zealand white rabbits (Laboratory Animal Centre of Hainan Medical College, Hainan, China) were used. The rabbits were about 12 ± 1 months old and their average weight was 3.5 ± 0.5 kg.

### Experimental design

The rabbits (*n* = 28) were randomly divided into four experimental groups (each group *n* = 7): sham group (no implantation), isolated α-CSH (Trauma Laboratory, Hainan, China) group, isolated ACB group and α-CSH/ACB mix group (α-CSH 50%, ACB 50%).

### Surgical procedure

**Fig. 1:**
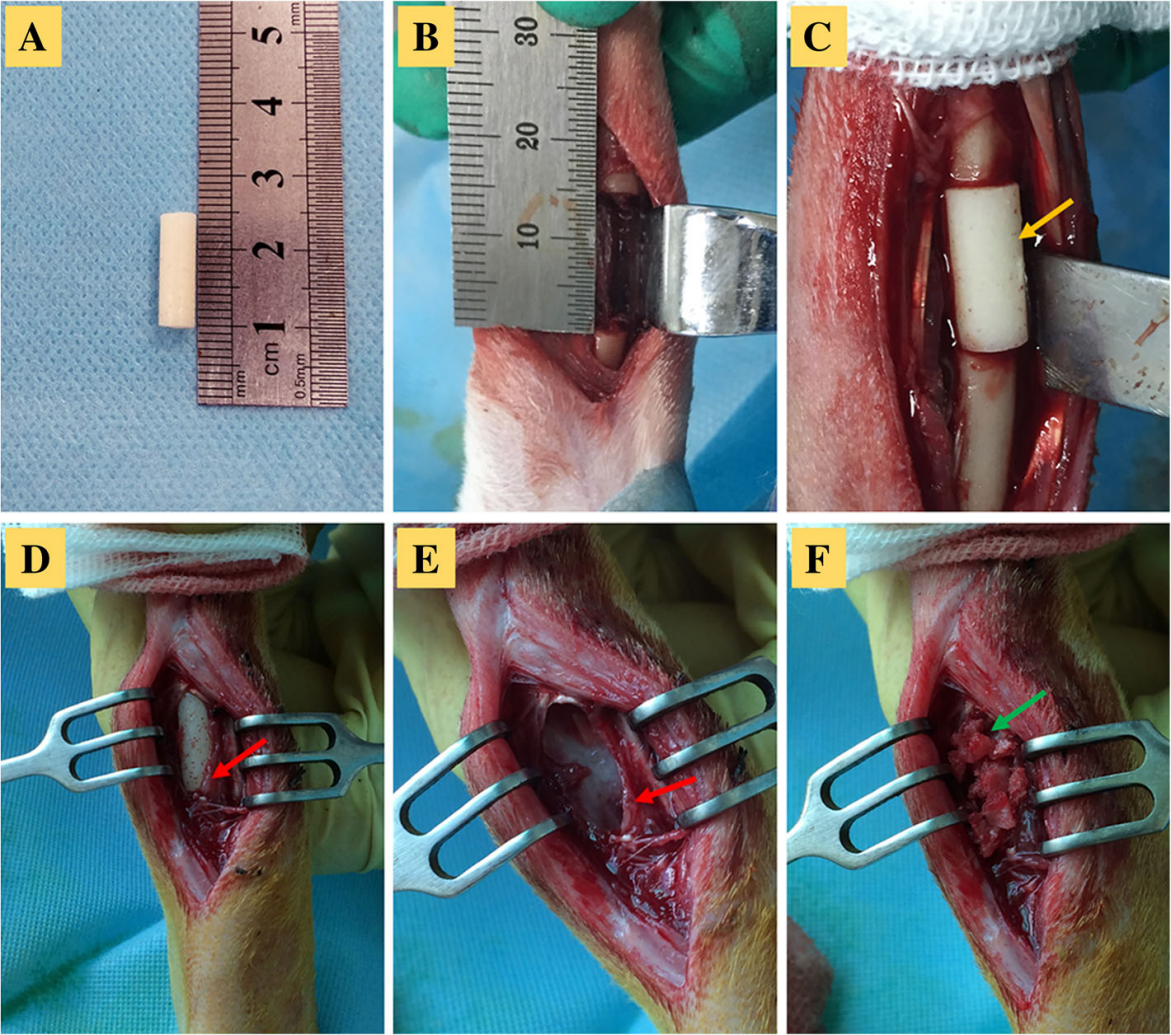
Intraoperative photographs of the Masquelet technique. **a** The PMMA spacer molded in vitro. **b** Creation of a critical-sized defect. **c** Insertion of the PMMA spacer (yellow arrow) into the defect. **d** Formation of the induced membrane (red arrow). **e** Removal of the PMMA spacer and formation of a cavity. **f** Placement of an autologous cancellous bone graft (green arrow) into the cavity

The rabbits were given general anesthesia by intravenous injection of 10% (*w*/*v*) chloral hydrate (3 ml/kg; Solarbio, Beijing, China). Additional regional anesthesia of the brachial plexus with lidocaine hydrochloride was carried out before the osteotomies. The rabbits were positioned in the supine position, and the left limb was prepared with povidone-iodine. A 4-cm longitudinal incision was created along the lower third of the forearm and a 15-mm critical-sized defect was then created in the left distal radius (Fig. 1b). A cylindrical PMMA spacer (Fig. 1a, 4.5-mm in diameter, 15-mm in length, Simplex P; Kalamazoo, MI, USA) was shaped in vitro and placed into the bone defect (Fig. 1c). The wound was closed with rapidly absorbable 4/0 subcutaneous (Jinhuan, Shanghai, China) and cutaneous sutures and then fixed with a small splint. The second surgical step was performed 6 weeks after the first procedure, under similar anesthetic and experimental conditions. The induced membrane was longitudinally opened and carefully elevated (Fig. 1d). The PMMA spacer was then carefully removed (Fig. 1e). Autogenous cancellous bone was harvested from the iliac crest during the same operation. Then, the cavity delineated by the induced membrane was grafted in line with the experimental design (Fig. 1f). The incised edges of the membrane surrounding the cavity and the muscles were loosely apposed and sutured by simple continuous with rapidly absorbable 4/0 sutures. Cefazolin sodium (100 mg/kg) was administered intramuscularly at 0, 24, and 48 h postoperatively. The operated limb was immobilized for 6 weeks using a small splint.

### Radiography

After general anesthesia by intraperitoneal injection of 10% (*w*/*v*) chloral hydrate (3 ml/kg), anteroposterior and lateral radiographs of the left radius (seven animals per group) were obtained using a portable X-ray machine (40 kV and 6.3 mA; Carestream Health, Inc., Rochester, NY, USA) and digital plates (Agfa; Scoresby VIC, Australia) at 4, 8, and 12 weeks after the second operation. The radiographs were scored using the Lane–Sandhu scoring system (Table 1). Scoring was carried out by three experts in the department of imagology, who was blind to the status of the rabbits.

**Table 1.** Lane–Sandhu radiographic scoring standard

Category	Standard	Scores
Callus	no callus	0
	callus occupying 25% of defect	1
	callus occupying 50% of defect	2
	callus occupying 75% of defect	3
	callus occupying 100% of defect	4
Fracture line	Clear	0
	Relatively clear	1
	Partial fracture line	2
	Basically vanished	3
	Completely vanished	4
Bone remodeling	No bone remodeling	0
	Remodeling of the intramedullary channel	2
	Full remodeling of cortex	4

### Three-dimensional reconstruction of spiral computed tomography

After general anesthesia by intraperitoneal injection of 10% (*w*/*v*) chloral hydrate (3 ml/kg), the rabbits (seven animals per group) were positioned in the prone position and scanned using a spiral-computed tomography (CT) multidetector at 12 weeks after the second operation. Using a middle frequency kernel, 1-mm-thick axial images underwent three-dimensional (3D) multiplanar reconstruction using the OsiriX MD image processing software (Pixmeo, Geneva, Switzerland) and used to quantify the volume of newly formed bone in the defect. These results were analyzed by a workstation (Philips, Amsterdam, Netherlands) by the same operator blinded to the status of the rabbits.

### Histological analysis

Twelve weeks after the second operation, the rabbits were killed and the samples were collected using a precision saw (Buehler Corporation, Lake Bluff, IL, USA). The samples were fixed in 4% paraformaldehyde for 48 h, decalcified in 10% (*w*/*v*) EDTA (pH 7.2–7.4; Solarbio, Shanghai, China) at room temperature for 3 weeks [28], washed with running tap water (purified) overnight, and dehydrated in graded ethanol and embedded in paraffin. Then, the samples were cut into 5-μm-thick microsections in the sagittal plane and stained with Hematoxylin and Eosin (H&E) staining and Masson’s trichrome staining (Solarbio, Shanghai, China), and observed under a light microscope (Nikon, Tokyo, Japan) and photographed (Nikon, Tokyo, Japan). The histological analysis was performed using Lane–Sandhu histological score standard (Table 2). Scoring was carried out by three experts in the department of pathology who was blind to the status of the rabbits.

**Table 2.** Lane–Sandhu histological score standard

Category	Standard	Scores
Union	No sign of union	0
	Fibrous union	1
	Osteochondral union	2
	Bone union	3
	Complete reorganization	4
Spongiosa	No sign of cellular activity	0
	Early bone formation	1
	Active new bone formation	2
	Reorganized spongiosa formation	3
	Complete reorganized spongiosa	4
Cortex	Absence of cortex	0
	Early detection	1
	Initiation of formation	2
	Reorganization in majority	3
	Complete organization	4

### Alkaline phosphatase assay

Alkaline phosphatase (ALP) activity was detected by BCIP/NBT alkaline phosphatase color development kit (Beyotime, Shanghai, China) in the bone histological sections. The sections were first deparaffinized in the xylene and rehydrated in graded ethanol and washed with phosphate buffered saline (PBS) three times. A primary polyclonal anti-ALP antibody (Abcam, Cambridge, MA, USA) was used at a 1:200 dilutions and incubated overnight at 4 °C, and then incubated with BCIP/NBT mix detecting reagent for 24 h at 4 °C. These slides observed under a light microscope and photographed. Semi-quantitative analysis of ALP express was performed using Image-pro Plus software (Media Cybernetics Inc., Silver Spring, CO, USA)

### Immunofluorescence staining

Immunofluorescence staining was performed to detect the levels of osteocalcin (OCN) expression in the bone histological sections. The sections were first deparaffinized in the xylene and rehydrated in graded ethanol and washed with PBS three times. A primary polyclonal anti-OCN antibody (Abcam, Cambridge, MA, USA) was used at a 1:100 dilutions and incubated overnight at 4 °C, followed by incubation with Alexa Fluor488-conjugated donkey polyclonal secondary antibody (Abcam, Cambridge, MA, USA) at a 1:1000 dilutions at 37 °C for 1 h. The slides were observed by laser scanning confocal microscopy (LSCM, Nikon, Tokyo, Japan). Semi-quantitative analysis of OCN express was performed using Image-pro Plus software.

### Statistical analysis

The data were expressed as the means ± standard deviation (SD). Statistical analysis was performed using one-way ANOVA and Student’s *t* test using SPSS statistical software 19.0 (SPSS Inc., Chicago, IL, USA). Statistical significance was determined at the level of *P* < 0.05.

## Results

All surgeries went well, and the rabbits recovered successfully from the operation and remained in good health. The implanted PMMA spacer and α-CSH did not cause any symptoms of infection or inflammation.

### Radiography

#### Sham group

**Fig. 2:**
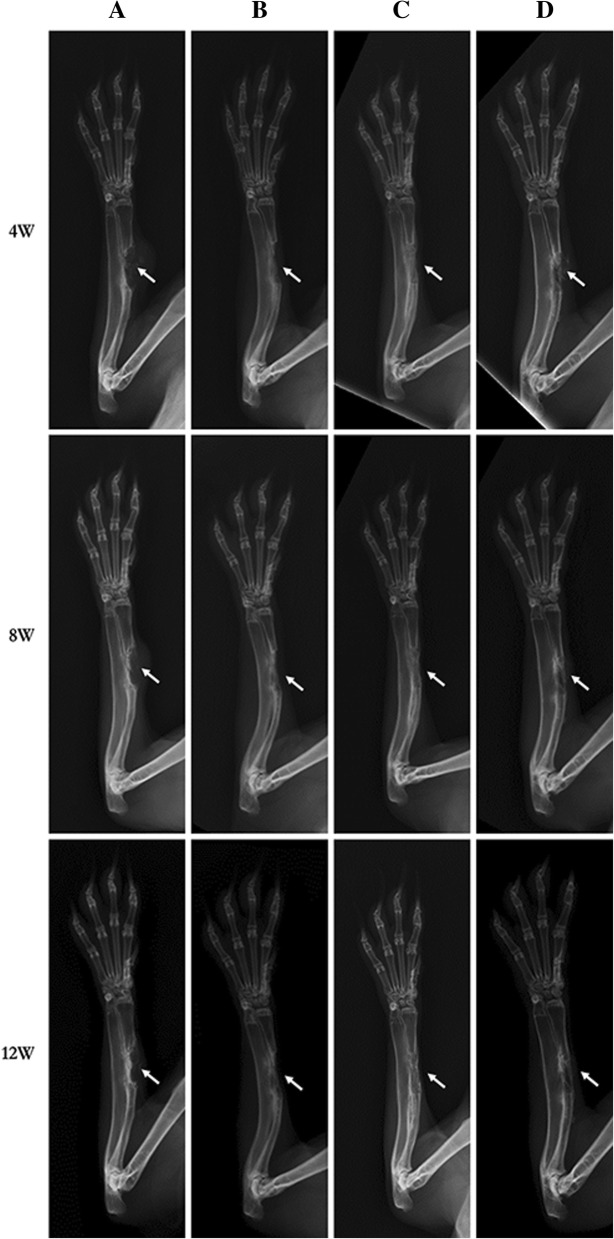
Radiographic assessment of bone formation. The representative radiographs of the bone mineral density in four groups at each time-point. The callus formation and union were observed by direct radiographs taken at 4, 8, and 12 weeks after the second operation. The white arrows over the images indicate the region of interest. **a** Sham group, **b** isolated α-CSH group, **c** α-CSH/ACB mix group, **d** isolated ACB group, *n* = 7

As the results shown (Fig. 2a), bone regeneration in sham group animals was limited to the vicinity of the cut edges of the defects. No bony union was observed radiographically in any animal.

#### Isolated α-CSH group

By 12 weeks, complete resorption of the plain α-CSH had occurred but this was not accompanied by sufficiently high rates of new bone formation to bridge the gap in any animal (Fig. 2b). Despite some new bone formation, non-union occurred and the bone marrow cavity was not formed in all animals.

#### α-CSH/ACB mix group

As early as 4 weeks after implantation, radiographs showed resorption of the α-CSH in all animals; this process was complete by the end of 8 weeks. Compared to the isolated α-CSH group (Fig. 2B), new bone formation occurred in all the α-CSH/ACB mix group animals within 12 weeks (Fig. 2c). Sufficient bone had formed within 12 weeks to bridge the gap in all animals. The bone marrow cavity was most re-opened and most remodeling of the cortex was occurred (Fig. 2c).

#### Isolated ACB group

The radiopacity of the ACB grafts was initially weaker than the radiopacity of the rest of the radius, but the values increased during subsequent weeks, almost matching that of the host bone by the end of 12 weeks. By that time, the bone union had occurred in all animals. The bone marrow cavity was complete re-opened and full remodeling of cortex occurred (Fig. 2d).

**Fig. 3:**
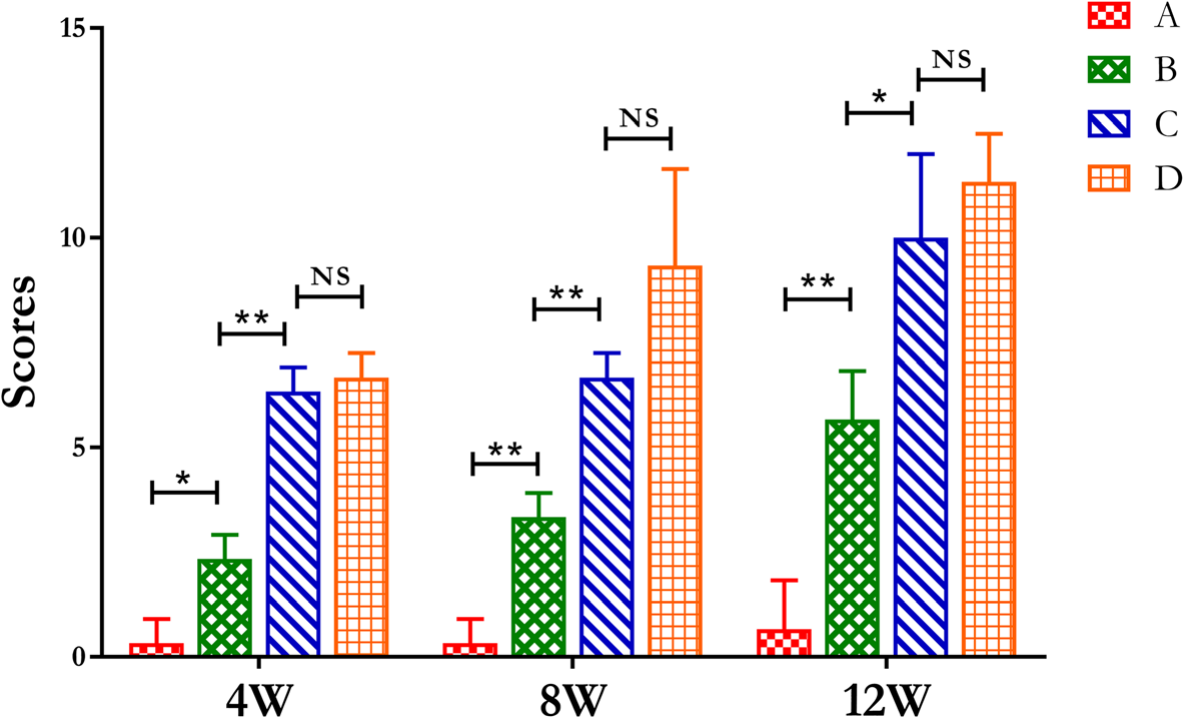
The radiographic scoring at 4, 8, and 12 weeks after the second operation assessed by the Lane–Sandhu scales. The scoring was obtained by averaging the scores from three different experts. **a** Sham group, **b** isolated α-CSH group, **c** α-CSH/ACB mix group, **d** isolated ACB group. Data represent mean ± SD. *P < 0.05; **P < 0.01; NS, not significant; *n* = 7

Radiological scores of the α-CSH/ACB mix group was significantly increased compared to the sham group and the isolated α-CSH group, while no significant difference compared to the isolated ACB group (Fig. 3).

### Spiral-CT

**Fig. 4:**
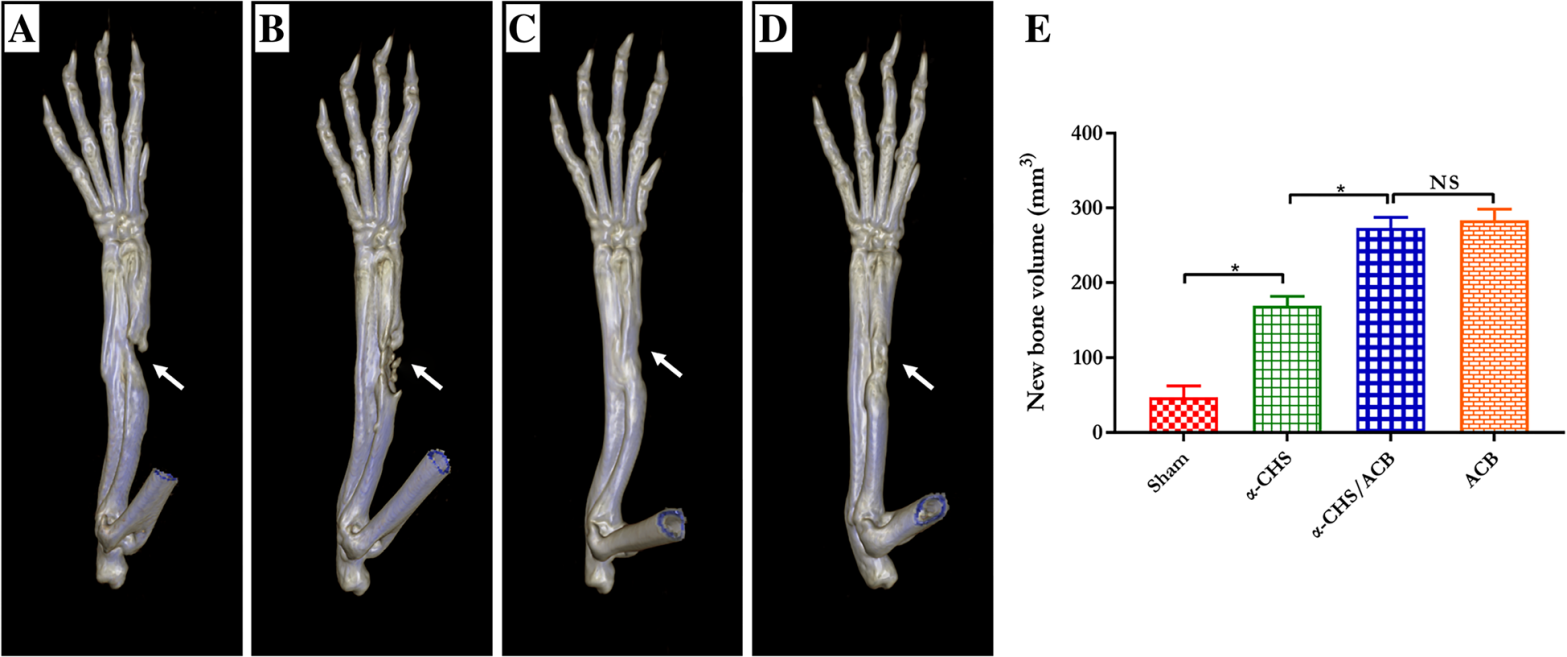
Three-dimensional reconstruction of spiral computed tomography analysis of bone formation at 12 weeks after the second operation. Representative 3D reconstruction of spiral-CT images from sham (**a**), isolated α-CSH (**b**), α-CSH/ACB mix (**c**), and isolated ACB group (**d**). Quantitative analysis of new bone volume (**e**). Data represent mean ± SD. *P < 0.05; NS not significant; *n* = 7

The 3D structures of the radius were reconstructed, and quantitative analysis was performed on a workstation (Volume Wizard; Philips). As the results shows (Fig. 4), newly formed bone volume in the isolated α-CSH group were significantly increased compared to the sham group. Newly formed bone volume in the α-CSH/ACB mix group was much higher than the isolated α-CSH group. However, there were no significant differences in the newly formed bone volume between the α-CSH/ACB mix group and the isolated ACB group.

### Histological analysis

**Fig. 5:**
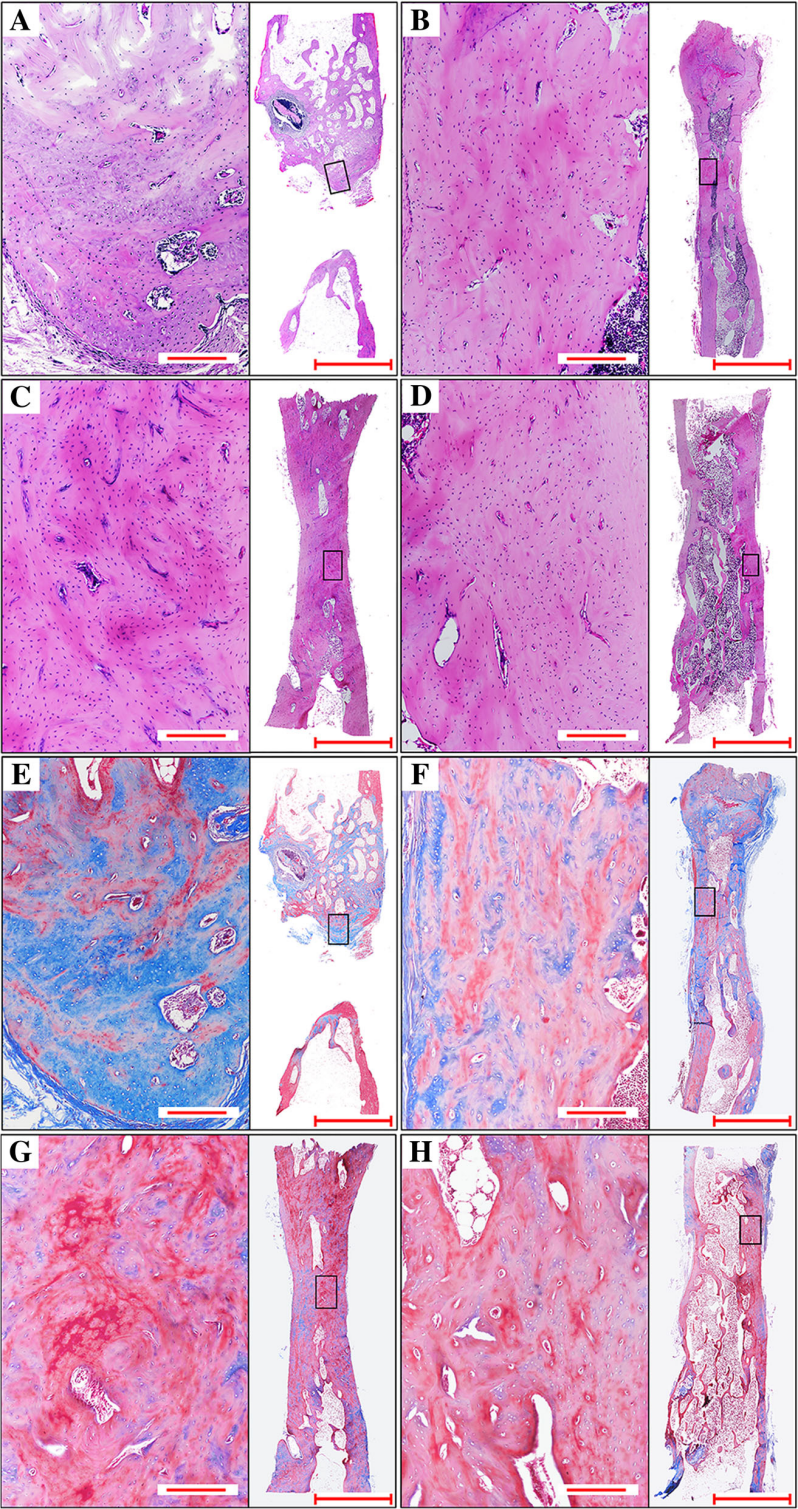
Histological and morphological analysis of bone formation. Representative HE stained (**a**–**d**) and Masson stained (**e**–**h**) images from sham (**a**, **e**), isolated α-CSH (B, F), α-CSH/ABC mix (**c**, **g**) and isolated ABC group (**d**, **h**). Boxed areas in the HE and Masson stained image at the right side (× 40) indicate the regions shown in the enlarged HE and Masson stained area at the left side (× 100). Scale bar = 6000 μm (× 40) and 900× μm (× 100)

**Fig. 6:**
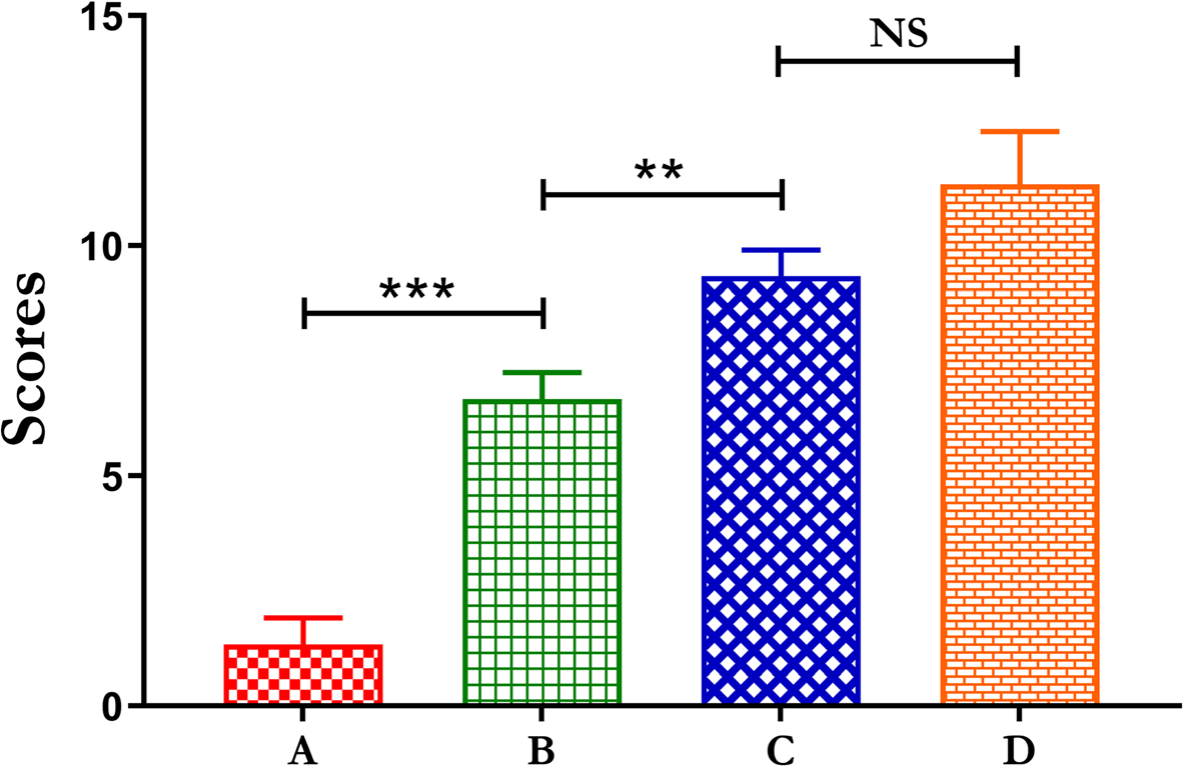
The histological scoring 12 weeks after the second operation assessed by the Lane–Sandhu histological score standard. The scoring was obtained by averaging the scores from three different experts. **a** Sham group, **b** isolated α-CSH group, **c** α-CSH/ACB mix group, **d** isolated ACB group. Data represent mean ± SD. **P < 0.01; ***P < 0.001; NS not significant; *n* = 7

To observe the histological and morphological characteristics, we examined the histological sections by H&E and Masson’s trichrome staining in all groups. As the results shows (Fig. 5), in the sham group, a few new bone was observed in the vicinity of the cut edges of the defects. In the isolated α-CSH group, the biomaterial was absorbed completely. The previous defects were filled with abundant new bone. The new bone displayed mostly blue collagenous fibers and the bone marrow cavity was not formed. In the α-CSH/ACB mix group, the biomaterial was absorbed completely. The volume of new bone is much more than that in the isolated α-CSH group and contained more red collagenous fibers than blue ones. The bone marrow cavity was most re-opened. In the isolated ACB group, the previous defects were filled completely with new bone and red collagenous fibers much more than the α-CSH/ACB mix group, which indicated that the new bone were undergoing gradual calcification and maturation. The bone marrow cavity was completely re-opened. Histological scores of the α-CSH/ACB mix group was significantly increased compared to the sham group and the isolated α-CSH group, while no significant difference compared to the isolated ACB group (Fig. 6).

### Alkaline phosphatase assay

**Fig. 7:**
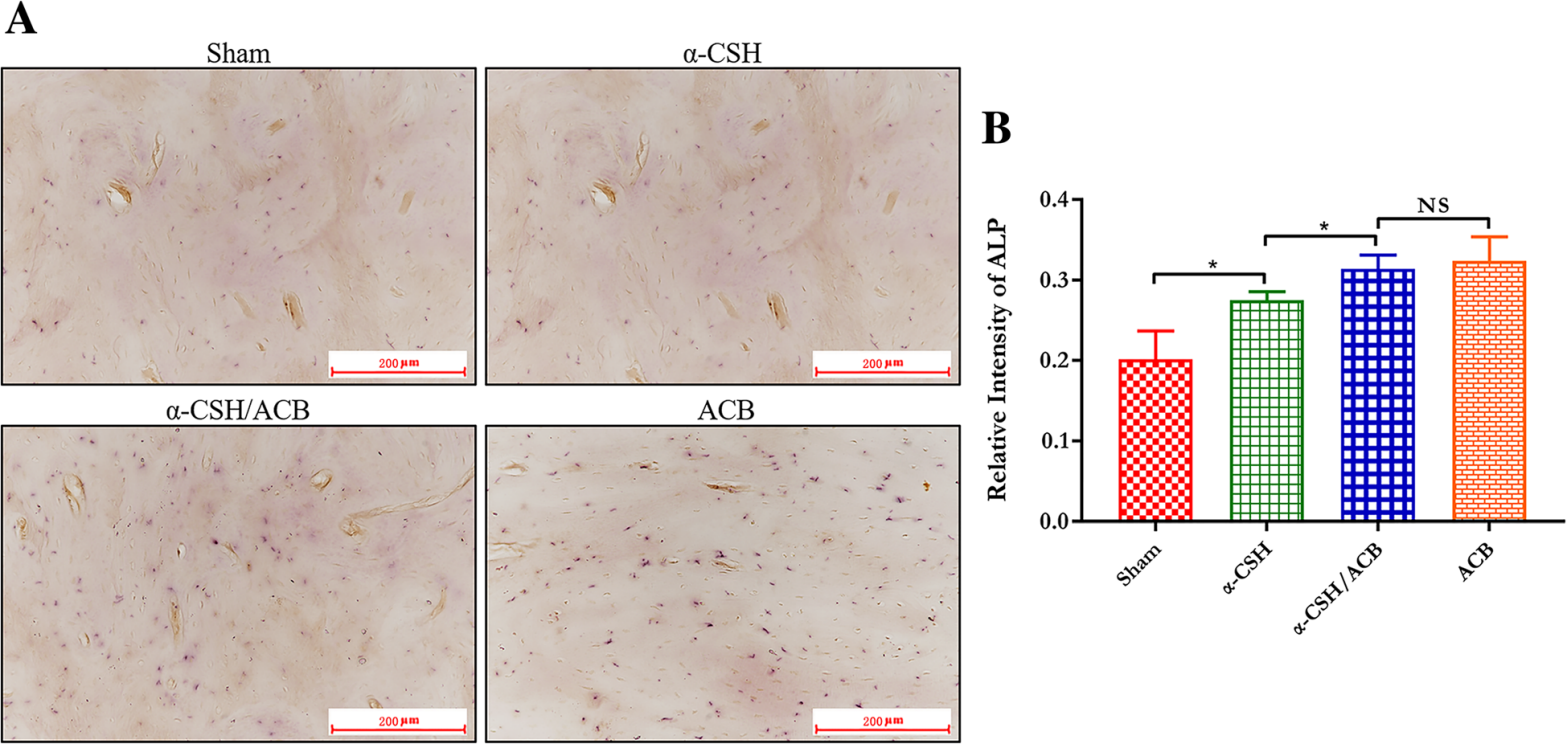
Immunohistochemical analysis of the levels of ALP expression. Representative images of immunostaining for ALP (**a**) from sham, isolated α-CSH, α-CSH/ACB mix, and isolated ACB group. Scale bar = 200 μm (× 100). Quantitative analysis of ALP expression (**b**). Data represent mean ± SD. *P < 0.05; NS not significant; *n* = 7

To determine the osteogenic activity of osteoblast, we examined the alkaline phosphatase (ALP) expression levels in all groups. As the results shown (Fig. 7), the levels of ALP expression were more prevalently expressed within the defects in the isolated α-CSH, α-CSH/ACB mix, and isolated ACB group compared to the sham group, and higher expression of ALP was observed in the α-CSH/ACB mix group and the isolated ACB group. However, there were no significant differences in the level of ALP between the α-CSH/ACB mix group and the isolated ACB group.

### Immunofluorescence staining

**Fig. 8:**
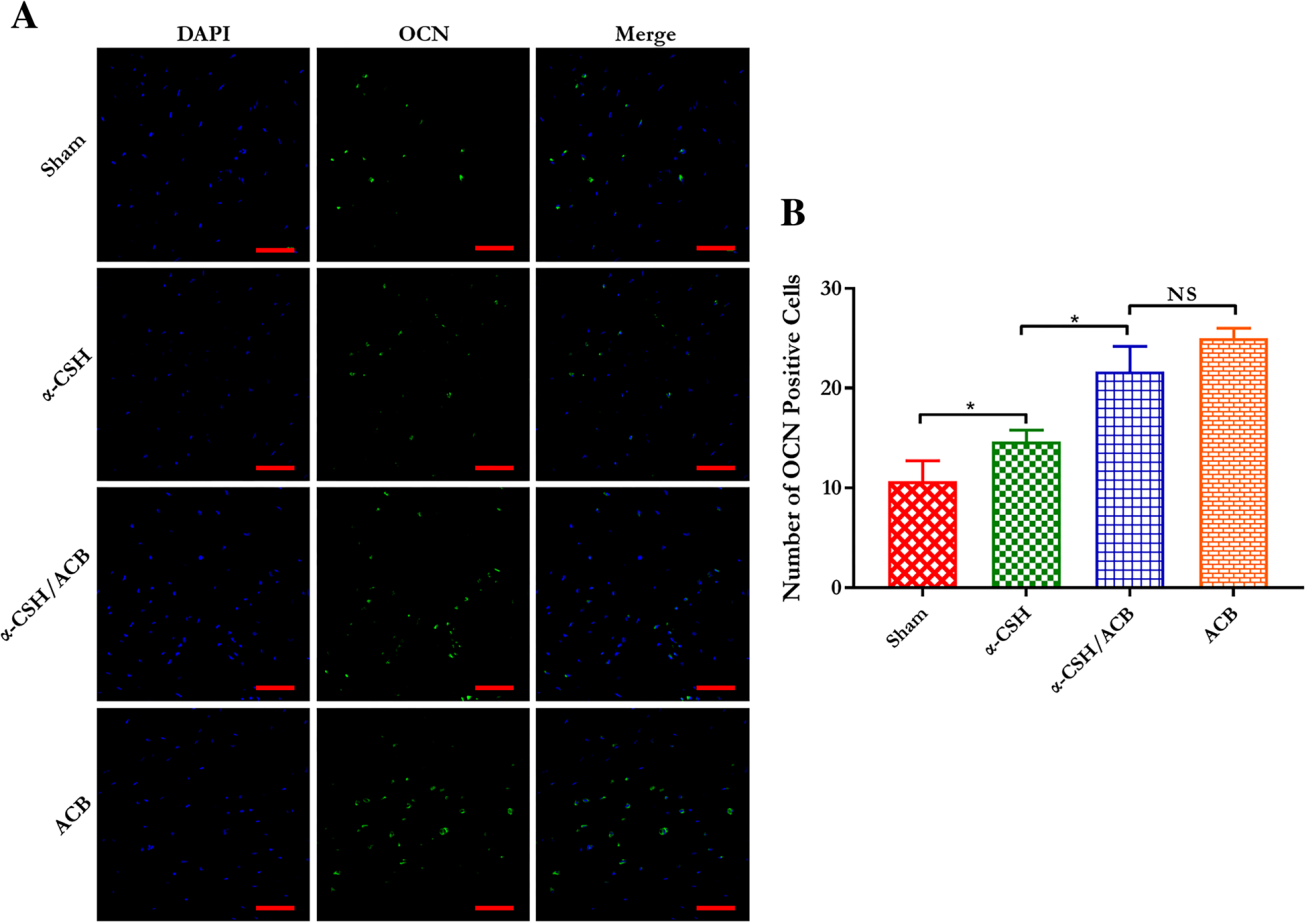
Immunofluorescence analysis of the levels of OCN expression. **a** Representative images of immunostaining for OCN from sham, isolated α-CSH, α-CSH/ACB mix, and isolated ACB group. Scale bar = 50 μm (× 400). **b** Quantitative analysis of OCN expression. Data represent mean ± SD. *P < 0.05; NS not significant; *n* = 7

To further determine the newly formed bone tissue, we examined the level of OCN expression by immunofluorescence staining. As the results shows (Fig. 8), the protein expression of OCN was significantly increased in three other groups compared to the sham group, and higher expression of OCN was observed in the α-CSH/ACB mix and isolated ACB group. However, there were no significant differences in the level of OCN between the α-CSH/ACB mix group and the isolated ACB group.

## Discussion

Reconstruction of large segmental bone defects remains a difficult challenge for orthopedic and trauma surgeons. Current solutions to manage large segmental bone defects include vascularized bone transfer and the Ilizarov intercalary bone method. However, these approaches have limitations because they are microsurgically and technically demanding. The Masquelet technique with ACB graft is a novel approach allowing reconstruction of large segmental bone defects [15, 29, 30]. Several research have highlighted the advantages of using the Masquelet technique: (1) maintain of ACB volume over time through protection of the ACB against resorption [4]; (2) restraint of the ACB in place [31]; (3) inhibit soft tissue invasion into the defect [18]; (4) produce growth factors and osteogenic factors, supporting the proliferation and differentiation of BMSCs [19, 32]. However, the Masquelet technique has limitations because of the supply of ACB and the complications of donor site [21–23]. Therefore, the use of bone substitute would be of great interest.

To the best of our knowledge, which is the most appropriate bone substitute remains controversial. Bone substitute must be selected for their properties to optimize bone repair, promote cell survival, proliferation, and differentiation, while exhibiting appropriate degradation rate, mechanical strength, excellent osteoconductivity, and osteoinductivity [33]. α-CSH is an outstanding bone substitute due to its easy availability, good biocompatibility, biodegradability, osteoconductivity, and a long history of use in bone reconstruction [34–36]. Moreover, it has been found to be potentially osteoinductive-like differentiation of BMSCs into osteoblasts [25]. A probable mechanism by which α-CSH can promote bone regeneration is by maintaining a high concentration of extracellular calcium ions, which is well known to have the effects of promoting osteoblast activity and inhibiting osteoclast activity [37–39]. However, the resorption rate of α-CSH is too fast to match the rate of new bone formation.

In this study, the combination of the Masquelet technique and isolated α-CSH or an α-CSH/ACB mix was investigated in a rabbit critical-sized defect model as they possessed the combined effects—sufficient supply of α-CSH and low resorption of the Masquelet technique. A practical definition of a critical-sized bone defect was suggested as a segmental bone defect of a length exceeding 2 to 2.5 times the diameter of the affected bone and will not heal spontaneously during the lifetime [17, 40]. To evaluate the optimal alternative solutions, we designed four groups: sham, isolated α-CSH, α-CSH/ACB mix (1:1, *w*/*w*), and isolated ACB group. The sham group is the negative control group to confirm that the critical-sized defect cannot heal spontaneously. The isolated ACB group is the positive control group to confirm that the critical-sized defect can heal using isolated ACB grafts. Our results showed that the sham group did not heal spontaneously. The Masquelet technique in conjunction with isolated α-CSH can partially heal critical-sized bone defect, while α-CSH/ACB mix (1:1, *w*/*w*) can approximately complete heal critical-sized bone defect. These results demonstrated that the Masquelet technique in conjunction with isolated α-CSH does not have enough capacity to repair a critical-sized defect. The bone repair capacity of α-CSH/ACB mix (1:1, *w*/*w*) is similar to isolated ACB (*P* > 0.05). This suggests that α-CSH can replace at least half of ACB, thus reducing the supply of ACB and the complication of donor site. In a further study, more ratio of α-CSH/ACB mixture should be investigated. In addition, α-CSH or α-CSH/ACB mix can be doped with osteogenic factors like bone morphogenetic protein (BMP) and vascular endothelial growth factor (VEGF) to further improve its ability to promote bone repair.

To further investigate the capacity of new bone formation of α-CSH at a molecular level, we examined the expression levels of ALP and OCN. Osteoblasts play a key role in bone formation [41]. ALP and OCN, known as the most abundant bone matrix enzyme and protein, are preferentially expressed by osteoblasts [42]. Therefore, ALP and OCN are often used as a typical biomarker for bone formation and maturation [43]. Our results showed that the ALP and OCN expression of the α-CSH/ACB mix group were higher than the isolated α-CSH group (*P* < 0.05) and were similar to the isolated ACB group (*P s*> 0.05). These results suggest that α-CSH and α-CSH/ACB mix can stimulate ALP and OCN expressions and then improve bone formation. In a further study, the definitive mechanism for stimulating ALP and OCN expression should be investigated.

In the first stage of the Masquelet technique, a PMMA spacer is inserted into the bone defect during surgery. In clinical practices, the spacer is molded in vivo. In this study, the spacer is molded in vitro. There is a shortcoming that the spacer does not completely match the diameter and length of the bone defect, which may affect the stabilization of the spacer and the formation of the induced membrane. In addition, another limitation of this study was the lack of an external fixation or a plate, as rabbit radius is too small to match the size of human external fixation and plate. Instead, a small splint fixation was used to enhance the stabilization of the PMMA spacer at the bone defect.

In the second stage of the Masquelet technique, we used α-CSH granules to fill the membranous tubes. In clinical practice, the shape and size of patients’ defects are highly variable. Standardized granules could fill defects of any size and shape and thus abolish the complex computer-assisted customization [44]. In a further study, which is the optimal size and shape of α-CSH or other bone substitute should be investigated.

## Conclusion

In conclusion, this study demonstrates that the Masquelet technique in conjunction with α-CSH/ACB mix (1:1, *w*/*w*) can complete repair rabbit critical-sized bone defect. When the amount of ACB is not sufficient in clinical practice, α-CSH might be a promising bone substitute to replace at least half of ACB to repair large segmental bone defect.

## Data Availability

The datasets used and/or analyzed during the current study are available from the corresponding author on reasonable request.

## Abbreviations

ACB: Autologous cancellous bone
ALP: Alkaline phosphatase
BMSCs: Bone mesenchymal stem cells
OCN: Osteocalcin
PBS: Phosphate buffered saline
PMMA: Polymethyl methacrylate
α-CSH: α-Calcium sulfate hemihydrate
